# Expression of PRB, FKBP52 and HB-EGF Relating with Ultrasonic Evaluation of Endometrial Receptivity

**DOI:** 10.1371/journal.pone.0034010

**Published:** 2012-03-20

**Authors:** Ning Wang, Linlin Geng, Shucheng Zhang, Bin He, Jiedong Wang

**Affiliations:** National Research Institute for Family Planning, Beijing, China; State Key Laboratory of Reproductive Biology, Institute of Zoology, Chinese Academy of Sciences, China

## Abstract

**Background:**

To explore the molecular basis of the different ultrasonic patterns of the human endometrium, and the molecular marker basis of local injury.

**Methodology/Principal Findings:**

The mRNA and protein expression of FKBP52, progesterone receptor A (PRA), progesterone receptor B (PRB), and HB-EGF were detected in different patterns of the endometrium by real-time RTPCR and immunohistochemistry. There were differences in the mRNA and protein expression of FKBP52, PRB, and HB-EGF in the triple line (Pattern A) and homogeneous (Pattern C) endometrium in the window of implantation. No difference was detected in PRA expression. After local injury, the mRNA expression of HB-EGF significantly increased. In contrast, there was no difference in the mRNA expression of FKBP52, PRB, or PRA. The protein expression of FKBP52, PRB, and HB-EGF increased after local injury. There was no difference in the PRA expression after local injury.

**Conclusions:**

PRB, FKBP52, and HB-EGF may be the molecular basis for the classification of the ultrasonic patterns. HB-EGF may be the molecular basis of local injury. Ultrasonic evaluation on the day of ovulation can be effective in predicting the outcome of implantation.

## Introduction

Ovarian estradiol (E_2_) and progesterone (P_4_) are the primary regulators of women's menstrual cycle [1]. In each cycle, the endometrium undergoes periodical changes to prepare for the implantation of competent blastocysts [2], [3]. To achieve successful implantation, the endometrium should be receptive and responsive to competitive embryos developing in a certain stage called “implantation window”. Thousands of proteins are involved in this process [4].

P_4_ plays a critical role in the female reproduction [2]. P_4_ need to bind with the specific progesterone receptor (PR) in order to activate the downstream regulated genes transcript involved in ovulation, endometrial receptivity, implantation, decidual reaction and the maintenance of pregnancy. PR can be classified into two isoforms, namely, PRA and PRB. PR is located in the eighth choromosome, and PRB is longer than PRA [5].

To play the role of transcriptional activation, PR needs to bind with a chaperone Fkbp52 during the process of implantation. Fkbp52 is an immune-affinity protein containing a tetratricopeptide repeat (TRP) domain that acts as a cochaperone with heat shock protein 90 (HSP90). FKBP52 plays a key role in the implantation of mammals, as confirmed by studies on fkbp null mice [6], [7].

HB-EGF is one of the accepted markers of endometrial receptivity. HB-EGF expression periodically changes in the different stages of a menstrual cycle. The changes are characterized by a low expression during proliferation, a gradual increase after ovulation, and an eventual increase to the peak at the time of implantation [8].

Non-invasive vaginal sonography has been used in clinical diagnosis for many years to evaluate the outcome of implantation [9], [10]. The ultrasonic patterns of endometria can be classified into two, namely, Pattern A and Pattern C.

Pattern A endometrium is a typical multi-layered “triple line” that consists of obvious outer and central hyperechogenic lines as well as inner hypoechogenic dark areas. Pattern C endometrium is entirely homogeneous and hyperechogenic. It is characterized by increased reflectivity compared with the myometrium and consequently has a brighter grayscale appearance. Its central hyperechogenic line is not visible. The pregnancy rate is significantly higher in the patients with Pattern A endometrium (30% per cycle) than in those with Pattern C endometrium (9.7% per cycle) [11]. This low pregnancy rate of the patient with Pattern C endometrium has been effectively treated in many in vitro fertilization (IVF) centers by adopting local injury on the day of ovulation before IVF application [12]–[15]. The reasons for endometrial ultrasonic assessment being able to predict the outcome of implantation and local injury being able to improve the success rate of implantation remain unknown.

The current study attempts to explore the molecular basis of the different ultrasonic patterns of the human endometrium, and the molecular basis of local injury. The protein and mRNA expression of PRA, PRB, FKBP52, and HB-EGF in Pattern A, Pattern C, and stimulated Pattern C (Pattern SC) endometria are reported.

## Results

### Baseline characteristics of Group A and Group C

The twenty women in the current study were divided into Group A and C. Group A had Pattern A endometrium and Group C had Pattern C endometrium. There was no difference between the two groups in their basal characteristics such as age, years of infertility, and menstrual cycle (P>0. 05), as shown in Table 1.

**Table 1:** The basal characteristics of Group A and Group C.

Group	N	Age (years)	Duration of Infertility (years)	Menstrual Cycle (days)
A	11	27.73±2.98	3.27±0.56	29.07±2.22
C	9	28.44±2.93	3.98±0.37	30.22±3.15

The serum level of E_2_ and P_4_ in Group A, C and SC were all within the normal range. There was no significant difference between Group A and C or Group C and SC (P>0. 05), as shown in Table 2.

**Table 2:** The serum level of E2 and P4 in Group A , C and SC in the window of implantation.

Group or Pattern	N	E2 (pmol/ml)	P(nmol/L)
A	11	154.29±1.57	17.49±2.46
C	9	188.82±1.61	18.85±1.43
SC	9	155.60±1.42	13.24±2.34

### The ultrasound image of Pattern A, C and SC endometrium

**Figure 1:**
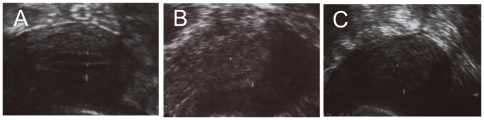
The ultrasonic image of Pattern A, C and SC endometrium.

Pattern A was typical triple-layer with central to the outer hyperechoic line between the outer and middle uterine hypoechoic areas or dark areas (Figure 1A). Pattern C was homogeneous hyperechoic mid-line cavity echo-free (Figure 1B). Pattern SC was an intermediate pattern with a weak and vague triple-line endometrium, with the central echogenic line nonprominent or absent (Figure 1C).

### Tissue morphology

Pattern A endometrium was easy to scrape, elastic, and shiny. Pattern C endometrium was thinner than those of Pattern A, less elastic and shiny, and mostly thin flakes. Pattern SC endometrium improved in thickness, elasticity and shine.

**Figure 2:**
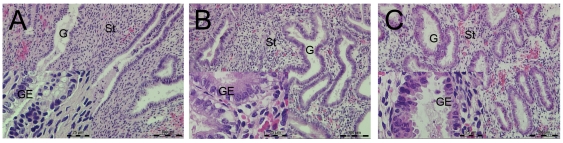
The HE staining of Pattern A, C and SC endometrium. St = stroma; G = gland; GE = glandular epithelium, 200×magnification.

Pattern A endometrium had typical mid-secretory features, rich secretions in gland cavity, jagged glands, stromal edema and had no mitotic figures, (Figure 2A). Sub-nuclear vacuoles were widely distributed in glandular epithelium and with mitotic figures occasionally noticed in Pattern C endometrium, which resembles the early secretory endometrium (Figure 2B). About 18.18% (2 out of 11) of the samples in Pattern A endometrium and 55.55% (5 out of 9) of the samples in Patter C endometrium had sub-nuclear vacuoles. After local injury, the gland was rich with secretion in Pattern SC endometrium,and the nuclear migrated to the bottom of the epithelium (Figure 2C). About 22.22% (2 out of 9) of the samples in Pattern SC endometrium had sub-nuclear in glandular epithelia.

### Real-time RT-PCR

**Figure 3:**
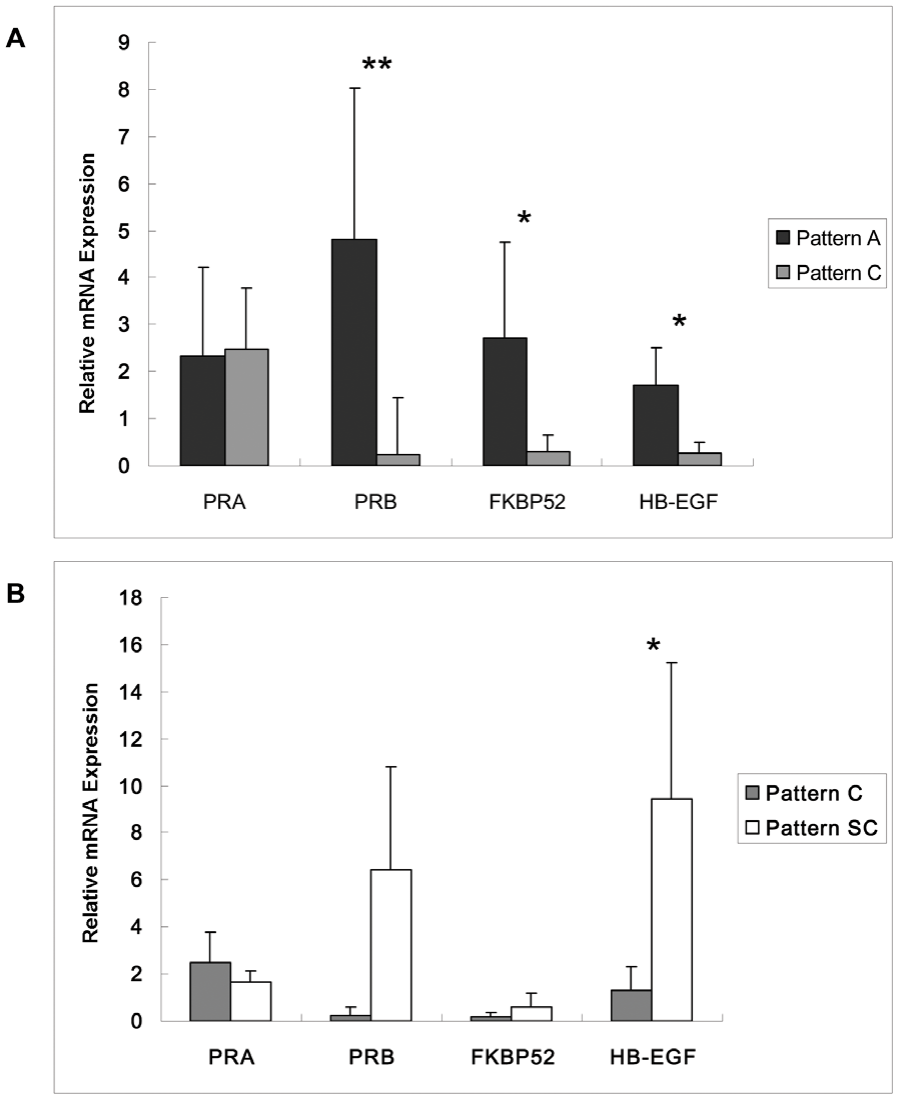
The mRNA expression pattern of PRA, PRB, FKBP52, and HB-EGF. The level of PRA, PRB, FKBP52, and HB-EGF mRNA was measured by real-time RT PCR. The values were normalized to GAPDH. A, the mRNA expression was compared between the patients with endometrium of Pattern A and Pattern C by Heteroscedastic T-test. B, the mRNA expression was compared between the same patient with endometrium of Pattern C and Pattern SC by paired T test. *, P<0.05; **, P<0.01.

The mRNA expression of FKBP52, PRB and HB-EGF was significantly higher in Pattern A endometrium than in Pattern C endometrium (P<0.05). There was no difference in the expression of PRA between the endometrium of two patterns (P>0.05), as shown in Figure 3A.

The mRNA expression of HB-EGF was significantly higher in Pattern C endometrium than in Pattern SC endometrium (P<0.05). There was no difference in the mRNA expression of FKBP52, PRB and PRA between the two patterns (P>0. 05), as shown in Figure 3B.

### Immunohistochemistry ( IHC) staining and H-score analysis

#### Distribution of PR and PRB in Pattern A, Pattern C and Pattern SC endometrium

**Figure 4:**
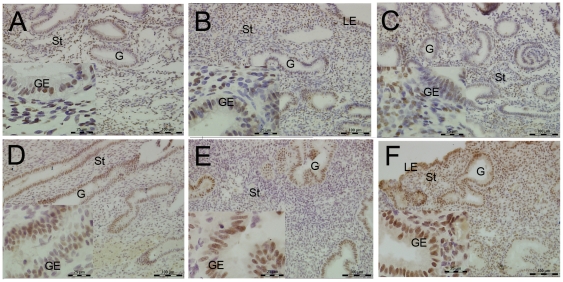
Immunolocalization of PRA and PRB in Pattern A, Pattern C and Pattern SC endometrium by IHC. A, B, and C were the staining of the PRA; D, E, and F were the staining of PRB. Positive staining of PRA changed insignificantly in different patterns of endometrium. Positive staining of PRB was weaker in Pattern C endometrium (E) compared with Pattern A endometrium (D), after locally injured, the PRB staining increased (F). St = stroma; G = gland; GE = glandular epithelium, 200×magnification.

**Figure 5:**
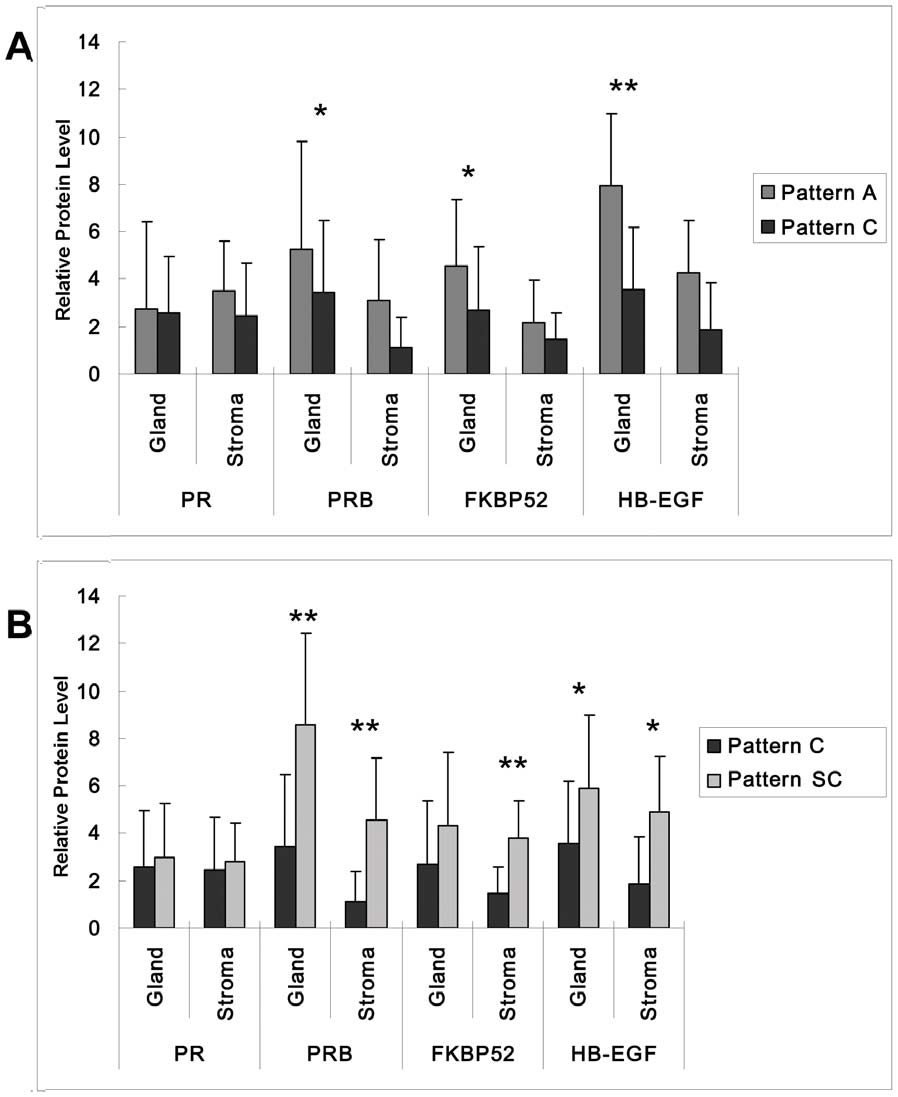
H-score analysis of PRA, PRB, FKBP52, and HB-EGF in three different pattern of endometrium. H-score was semiquantitatively come from the intensity and distribution of positive staining cells and represented the relative protein level. A, H-score was compared between Pattern A and Pattern C endometrium. B, H-score was compared between Pattern C and Pattern SC endometrium.

The protein expression of PR and PRB in the endometrium of different patterns was detected by IHC. The cellular distribution of PR was mainly in the nuclear area of stromal cells (Figure 4A to 4C). The H-score analysis of epithelial and stromal cells of PR showed no differences among the endometrium of the three patterns (Figure 5).

In Pattern A endometrium, the protein expression of PRB was mainly distributed in both the glandular epithelium and the stromal cell (Figure 4D). In Pattern C endometrium, the protein expression of PRB was mainly distributed in the glandular epithelium, with a small portion stained in the stromal cells (Figure 4E). However, the protein expression of PRB in Pattern SC increased rapidly both in the glandular and stromal cells after the endometrium was locally injured (Figure 4F). The H-score analysis of PRB showed a statistically significant difference in the stromal cells between Pattern A and C endometrium (Figure 5A, *P<0.05). The protein expression of PRB was significant higher in both epithelial and stromal cells in Pattern SC endometrium than in Pattern C endometrium (Figure 5B * *P*<0.05).

#### FKBP52 distribution in the endometrium of the three patterns

**Figure 6:**
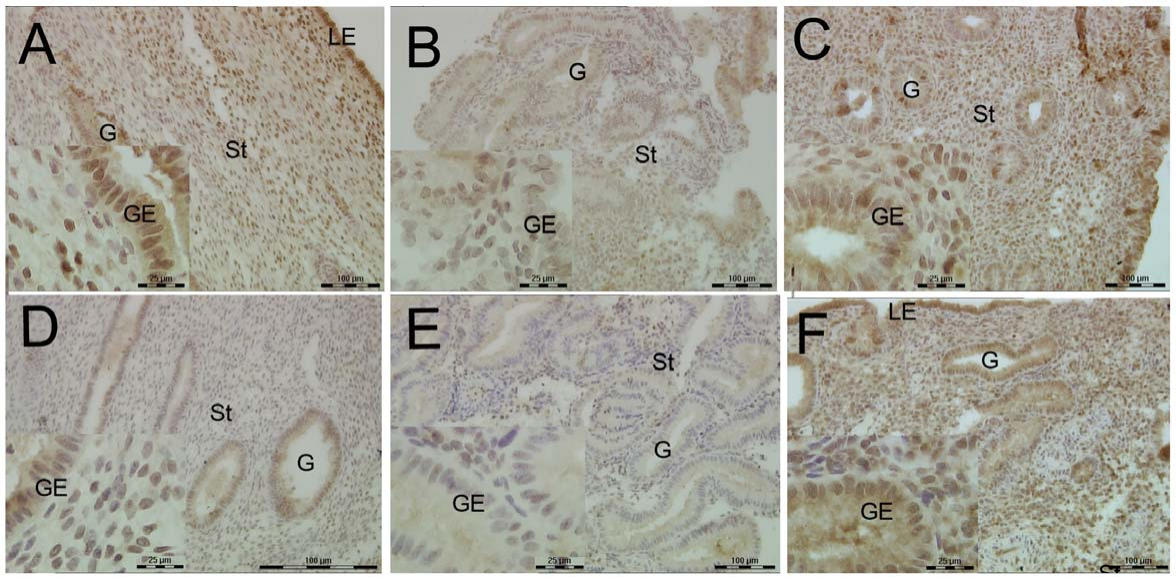
FKBP52 and HB-EGF distribution in the endometrium of the three patterns. A, B, and C were the staining of the FKBP52; D, E, and F were the staining of HB-EGF. Positive staining of FKBP52 and HB-EGF PRB was weaker in Pattern C endometrium (Band E) compared with Pattern A endometrium (A and D), after locally injured, the PRB staining increased (C and F). St = stroma; G = gland; GE = glandular epithelium; LE = luminal epithelium, 200×magnification.

The protein expression of FKBP52 was higher in Pattern A endometrium than in Pattern C endometrium (Figure 6A, 6B). Strong positive signals were located in the glandular epithelium of endometria, with faint staining in the stromal cells in Pattern A endometrium. Weak positive signals were detected in Pattern C endometrium (Figure 5A *P<0.05). The positive signals were mainly located in the nuclear and plasma of epithelia (Figure 6A, 6B). After local injury, the protein expression of FKBP52 increased significantly in stromal cells, and there were few changes in the epithelial cells of Pattern SC (Figure 6B, 6C and Figure 5B *P<0.05).

#### H-score analysis of HB-EGF protein in the three patterns endometrium

HB-EGF protein was mainly distributed in the plasma of gland epithelial and stromal cells. Consistent with the mRNA expression, HB-EGF was higher distributed in Pattern A endometrium than in Pattern C endometrium (Figure 6D, 6E and Figure 5A *P<0.05), specially in the epithelial cells of glands (Figure 5, **P<0.01). A comparison between Pattern C and SC endometrium reveals that the HB-EGF increased in the stromal cells of Pattern SC endometrium (Figure 6E, 6F and Figure 5B *P<0.05).

## Discussion

E_2_ and P_4_ are secreted by the ovaries and play important roles throughout the human menstrual cycle. The human endometrium has a strong ability to regenerate and thus plays a key role in the process of implantation [1]. In each menstrual cycle, the endometrium undergoes a series of cyclical changes in preparation for the implantation, with thousands of proteins involved in this process. Nevertheless, the human endometrium can be receptive to embryo implantation only within a short period of time during the menstrual cycles. This period is named “implantation window”, the endometrium is at the receptive state to ensure embryo implantation, fetal and placental development [16]. Ultrasound as a non-invasive method is effective in evaluating endometrial receptivity and thus predicting implantation outcome [11]. However, little is known about the factors that determine the ultrasonic pattern of endometrium.

After hematoxylin-eosin (HE) staining, there are differences in the development of endometrium depending on the patterns. The glandular and stromal cells were not fully developed in Pattern C endometrium, with some sub-nuclear vacuoles in glandular epithelia, which is the characteristic of early secretory phase. The nuclei migrated from gland lumen and the edema, as a characteristic marker of the mid-secretory phase, were obvious in the stroma of Pattern A endometrium. In contrast, less edema were observed in Pattern C endometrium. After endometrium was locally injured, the sub-nuclear vacuoles disappeared or decreased in the glands, and the epithelial gland of Pattern SC endometrium increased in secretion. The morphological differences between the two patterns can be caused by the difference in their responsiveness to P_4_. Local injury can be administered to improve the lag in the development of Pattern C endometrium [17].

PRB, instead of PRA, may be the molecular basis for the classification of the ultrasonic patterns. P_4_ plays an essential role in female reproduction [4]. The successful execution of the function of P_4_ is dependent on a specific nuclear receptor the PR [5].

In this study, no difference was observed in the expression of PRA between Pattern A and Pattern C endometrium. The same observation was made about the expression of PRA between Pattern C and Pattern SC endometrium. In the mid-secretory phase, the PR expression increased and was mainly located in the nucleus of the stromal cells, while the expression in the glandular epithelium declined sharply and ended up weak. These changes were detected in this study and there was no difference between Pattern A and Patter C or between Pattern C and Pattern SC. This finding verifies the consistency and accuracy of the endometrial samples. The PRB mRNA and protein expression were significantly higher in Pattern A endometrium than in Pattern C endometrium. The PRB differences between Pattern A and Pattern C endometrium were mainly in the stromal cells. These results were consistent with Mulac-Jericevic who suggested that PRB was critical in PR responsiveness to P_4_ [5]. In this study, the serum level of E_2_ and P_4_ were similar regardless of the endometrial pattern. In addition, there was no difference in PRA expression among the patients regardless of their endometrial pattern. However, the responsiveness of endometrium to P_4_ has great disparities among different patterns. Therefore, it was PRB that influenced the classification of the endometrium. When Pattern C endometrium was compared with Pattern SC endometrium, PRB protein was differentially expressed in both the glandular epithelial and the stromal cells. With Pattern C and Pattern SC as self-control of the same person and with no interferences from other individuals [18], PRB protein obviously played a role in improving the receptivity of Pattern C endometrium, since PRB protein can enhance the responsiveness of endometrial cells to P_4_.

The FKBP52 expression is also related to the classification of the endometrium. To execute the function of P_4_, PR binds with P4 and other components into a complex including the receptor, Heat Shock Protein (HSP) and cochaperone in the process of implantation [6], [7]. FKBP52, an immunophilin, contains a TRP repeat domain, which specifically binds with the highly conserved c-terminus of HSP90 and thus acts as a cochaperone with HSP90 [19]. Similar to other steroid receptors, PR assembles with chaperones in an ordered multi-step manner for binding hormones. The mRNA and protein expression of FKBP52 differed significantly between Pattern A and C endometrium. This finding indicated that FKBP52 may play an important role in classifying the endometrium of various ultrasonic patterns. The expression of FKBP52 in Pattern SC increased after local injury, which suggests that FKBP52 protein may play a role in improving the receptivity of the endometrium.

HB-EGF may be the molecular basis of local injury. HB-EGF expression is recognized as a marker of endometrial receptivity. HB-EGF is periodically expressed in the menstrual cycle of human. The expression of HB-EGF is low in the proliferative phase; it increased after ovulation and reaches the highest level at implantation [20], [21]. Once HB-EGF expression is started, cascade reactions are provoked. In the current work, the expression of HB-EGF increased significantly after the endometrium was locally injured. Highly expressed HB-EGF may play a paracrine role in the adjacent stromal cells, which caused extensive proliferation and differentiation [8]. Therefore, although the endometrium could not transform into a typical Pattern A endometrium after local injury, the cascade reactions resulted in numerous changes and eventually improved endometrial receptivity.

Various factors may account for the discrepancies between the expression of mRNA and protein levels. Firstly, the trends of mRNA and protein expression were consistent. The increase in protein was more obvious than that in mRNA. The former was statistically significant, while the latter was not. This discrepancy may be caused by the limited size of human samples in the present study. If the sample size is large enough, there is possible that the trend for the expression of RNA and protein levels will be consistent. Secondly, the FKBP52 mRNA expression was detected in the whole endometrial sample, whereas the significant change in FKBP52 protein can be seen only in the grand epithelia. In other words, the change in part of the protein level cannot reflect that of the whole mRNA sample.

In summary, PRB, FKBP52 and HB-EGF are more highly expressed in Pattern A endometrium than in Pattern C endometrium, which partly accounts for the higher pregnancy rate in patients with Patter A endometrium than in those with Pattern C endometrium. This study also suggests that PRB, FKBP52 and HB-EGF may be the molecular basis for the classification of the ultrasonic patterns. Local injury increased the protein expression of PRB, FKBP52 and HB-EGF; however, among the three factors, only HB-EGF underwent a significant increase in its mRNA expression, while no obvious changes were observed of PRB and FKBP52. Therefore, HB-EGF may be the molecular basis of local injury. In conclusion, because the pattern of endometrium on the day of ovulation is closely associated with the expression of PRB, FKBP52 and HB-EGF in the window of implantation, the ultrasonic evaluation of endometrium on the day of ovulation can be effective in predicting the outcome of implantation.

## Materials and Methods

### Study design and patients

Twenty women sought treatment in the assisted reproduction center of National Research Institute for Family Planning (NRIFP) of China from July to December in 2010. All reported unsuccessful pregnancy as a result of male infertility. The average age of the patients was 28.52 years old. The average duration of infertility was 3.50 years. Institutional review board approval was obtained from the Academic Committee of the National Research Institute for Family Planning on the use of Human Subjects in Medical Research. All the patients provided written informed consent.

### Sample collection and hormonal measurement

Twenty women were divided into two groups Group A and Group C. Group A had Pattern A endometrium as detected by an ultrasound scan. The samples of Pattern A endometrium were collected seven days after ovulation in the spontaneous menstrual cycle. Group C had Pattern C endometrium. The samples of Pattern C endometrium were collected seven days post ovulation when the endometrium was locally injured by curette. In the second spontaneous menstrual cycle, endometrial samples were collected again and the endometrium was named stimulated Pattern C (Pattern SC). Thus, the endometria of Pattern C and Pattern SC were self-controlled. Endometrial biopsies were taken from the anterior wall of the uterine cavity. Each biopsy was divided into two parts. One part of the harvested endometrial pieces was snap frozen in liquid nitrogen and then was stored at −70°C for no longer than six months. The other part was fixed in 4% paraformaldehyde for histochemical analysis. Blood samples were collected on the same day. Serum was separated and stored for the measurement of E_2_ and P_4_.

The serum level of E_2_ and P_4_ was measured by the enzyme-linked fluorescence assay (ELFA) as described previously [22], [23]. Briefly, the assay principle combines an immunoassay competition method with a final fluorescent detection. The solid phase receptacle (SPR) is coated with polyclonal rabbit anti-estradiol antibodies (PI- 30431, BioMérieux SA, Lyon, France) and monoclonal mouse anti-progesterone antibodies (PI-30409, BioMérieux SA, Lyon, France), respectively. The sample is transferred to a well containing E2 or PRG derivative labeled with alkaline phosphatase, which catalyzes the hydrolysis of 4-methyl-umbelliferyl phosphate into a fluorescent product. The fluorescent concentration and intensity is inversely proportional to the antigen in the sample at 450 nm.

### RNA isolation and real-time RT-PCR analysis

Endometrial samples were collected and stored in liquid nitrogen for RNA isolation. Trizol (Invitrogen) was used to isolate total RNA from human tissue according to manufacturer's protocol. Total RNA was quantified by UV spectrophotometry. One microgram of total RNA was reverse-transcribed by using the GeneAmp RNA PCR system 9700 (Applied Biosystems, Foster City, CA). The final reaction volume was 20 µl with 0.5 ug/µl Oligo (dT) 18. T he reaction conditions were 5 min at 65°C, 1 min at 37°C, 60 min at 50°C, and 15 min at 70°C. Real-time PCR was performed by using the following primer sequences:

PRA: F-GAGCCCACAATACAGCTTCGAG, R-CGAAAACCTCCAAGGACCATG;

PRB: F-GGCAGATGCTGTATTTTGCACC, R-CAAACCAATTGCCTTGATGAGC;

FKBP52: F-CATTGCCATAGCCACCATGAA, R-TCCAGTGCAACCTCCACGATA; HB-EGF: F-CTTTCTGGCTGCAGTTCTCTCG, R-GCCCCTTGCCTTTCTTCTTTC.

After 3 min of incubation at 95°C, 40 cycles were performed as follows: denaturation at 94°C for 20 s, annealing at 59°C for 20 s and extension at 72°C for 30 s. The results are normalized to the amount of GAPDH and expressed as abundance by the ΔCt method [24].

### Histology and IHC

The endometrial samples were processed by conventional preparation for histology and cut into 5 µm sections. HE staining was used to histological evaluate of the endometrial biopsies according to the criteria of Noyes et al [25].

For IHC, mouse anti-human PR (1A6) (NCL-PGR, Leica, USA) antibody was diluted 1∶ 200 in antibody dilution, and the rabbit anti human PRB (MS-192-P1, Thermo Scientific, UK) was diluted at 1∶250. The FKBP52 antibody (ab84536, Abcam, UK) was diluted at 1∶300, and the HB-EGF (AF-259-NA, R&D) was diluted at 1∶200. Sections were deparaffinized, rehydrated and washed in 0.1 M PBS (pH 7.4). The sections were then immersed in 3% hydrogen peroxide to block endogenous peroxidase and incubated for 5 to 10 minutes. Slides were incubated in antibodies against human PR, PRB, HB-EGF and FKBP52 at 4°C overnight. Signal was detected by adding biotinylated secondary antibodies and streptavidin-peroxidase, and stained using 3, 3′-diaminobenzidine plus peroxide solution.

### H-score analysis of immunostaining

The IHC staining of the four detected factors (PR, PRB, HB-EGF and FKBP52) was scored semiquantitatively by using the quick score method as described [26]. Both the intensity and distribution of the positive staining cells in all slides were measured blinded by an experienced pathologist.

### Statistical analysis

Average mean analysis was determinate by student's T test using the computer program SPSS 11.5. A value of P<0.05 was considered statistically significant.
